# Analysis of the Mitochondrial Dynamics in NAFLD: Drp1 as a Marker of Inflammation and Fibrosis

**DOI:** 10.3390/ijms26157373

**Published:** 2025-07-30

**Authors:** Maël Padelli, Jocelyne Hamelin, Christophe Desterke, Mylène Sebagh, Raphael Saffroy, Claudio Garcia Sanchez, Audrey Coilly, Jean-Charles Duclos-Vallée, Didier Samuel, Antoinette Lemoine

**Affiliations:** 1 Service de Biochimie et Oncogénétique, Hôpital Paul Brousse, Assistance Publique-Hôpitaux de Paris (AP-HP), Université Paris Saclay, 94800 Villejuif, France; jocelyne.hamelin@aphp.fr (J.H.); raphael.saffroy@aphp.fr (R.S.); claudio.garciasanchez@aphp.fr (C.G.S.); antoinette.lemoine@aphp.fr (A.L.); 2 Faculté de Médecine du Kremlin Bicêtre, Université Paris-Saclay, INSERM UMRS-1310, 94276 Le Kremlin-Bicêtre, France; christophe.desterke@inserm.fr; 3 Laboratoire d’Anatomopathologie, Hôpital Paul Brousse, Assistance Publique-Hôpitaux de Paris (AP-HP), 94800 Villejuif, France; mylene.sebagh@aphp.fr; 4 UMR-S 1193, Inserm, Physiopathogénèse et Traitement des Maladies du Foie, 94800 Villejuif, France; audrey.coilly@aphp.fr (A.C.); jean-charles.duclos-vallee@aphp.fr (J.-C.D.-V.); didier.samuel@aphp.fr (D.S.); 5 Centre Hépatobiliaire, Hôpital Paul Brousse, Assistance Publique-Hôpitaux de Paris (AP-HP), Université Paris Saclay, 94800 Villejuif, France

**Keywords:** non-alcoholic fatty liver disease, non-alcoholic steatohepatitis, fibrosis, cirrhosis, mitochondrial dynamics, mitochondrial fission, mitochondrial fusion, mitophagy, Drp1, Kupffer cells

## Abstract

Non-alcoholic fatty liver disease (NAFLD) is the most common chronic liver disease, projected to affect 55% globally by 2040. Up to one-third of NAFLD patients develop non-alcoholic steatohepatitis (NASH), with 40% progressing to fibrosis. However, there are currently few reliable tools to predict disease progression. Impaired mitochondrial dynamics, characterized by dysregulated fission, fusion, and mitophagy, have emerged as key events in NAFLD pathophysiology, contributing to hepatocyte death and inflammation. This study explored the transition from steatosis to NASH through transcriptomic analyses, including data from patients with steatosis and those with NASH at different fibrosis stages. By identifying a transcriptomic signature associated with disease progression, the study revealed increased expression of genes involved in mitochondrial dynamics in NASH compared to steatosis and during NASH-related fibrosis. Histological analyses highlighted the central role of Dynamin-related protein 1 (Drp1), a dynamin GTPase essential for mitochondrial fission and mitophagy. In human liver biopsies, Drp1 expression progressively increased from NAFLD to NASH and NASH-related fibrosis and cirrhosis, predominantly in Kupffer cells. These finding suggest Drp1 is a potential driver of the transition to more severe liver damage, making it a promising biomarker for NASH development and progression and a potential therapeutic target in metabolic disorders.

## 1. Introduction

Non-alcoholic fatty liver disease (NAFLD) is a chronic liver disorder marked by the accumulation of lipids within liver cells [1,2]. It includes a spectrum of hepatic conditions characterized by abnormal fat deposition in the liver without a history of excessive alcohol consumption. The disease encompasses a range of pathological states such as steatosis, steatohepatitis, fibrosis and cirrhosis, all of which are associated with a higher risk of hepatocellular carcinoma and significant extrahepatic complications [3,4]. NAFLD has become a major public health concern in Western nations due to its connection with metabolic syndrome, affecting approximately 20% of the general population and up to 90% of obese individuals undergoing bariatric surgery [5].

Hepatic steatosis is characterized by fat accumulation in more than 5% of hepatocytes. It is considered as a potential precursor to non-alcoholic steatohepatitis (NASH), a condition marked by steatosis, inflammation, and hepatocyte damage which can progress to cirrhosis or liver failure [6]. The pathogenesis of the transition from steatosis to NASH remains unclear, and reliable biomarkers for this process are currently unknown.

Evidence is accumulating suggesting that the onset of fatty liver disease is linked to impaired quality control of damaged mitochondria [7,8,9]. As central regulators of cellular function, mitochondria control essential processes, including oxidative stress modulation, ATP production, programmed cell death, and calcium signaling [10]. To maintain cellular integrity, isolated mitochondria continuously divide and fuse to form an interconnected network throughout the cell. An imbalance in these processes can be detrimental to mitochondrial function, leading to decreased respiration, increased reactive oxygen species (ROS) production, and apoptosis [11,12]. Impaired mitochondrial dynamics and mitophagy, the process by which damaged or dysfunctional mitochondria are selectively degraded, may be key events in the pathophysiology of NAFLD. Disturbances in these processes may facilitate the progression from simple steatosis to more severe liver damage.

This study explores the transition from steatosis to NASH through transcriptomic analyses performed on databases, including patients with steatosis and those with NASH at different fibrosis stages. By identifying a transcriptomic signature associated with disease progression, the study provides insights into key molecular changes in mitochondrial dynamics. Additionally, histological analyses were conducted to determine the tissue expression of mitochondrial dynamics proteins in order to better understand their role in NAFLD. The findings reveal an increase in mitochondrial dynamics, including fission, fusion, and mitophagy, in NASH compared to steatosis. Among these processes, Dynamin-related protein 1 (Drp1), essential for mitochondrial fission, stands out as a potential driver of the transition to more severe liver damage. Thus, Drp1 emerges as a promising biomarker for the development and progression of NASH in metabolic disorders, as well as a potential therapeutic target.

## 2. Results and Discussion

### 2.1. Mitochondrial Dynamics of Differentially Expressed Genes in NASH Compared to Steatosis

To investigate mitochondrial dynamics in NAFLD, a PubMed curation and a query of the Gene Ontology database were conducted, revealing representative gene sets for analyzing mitochondrial fission, fusion, and mitophagy functions. These functions were categorized into three distinct gene sets using the training dataset GSE48452.

**Figure 1:**
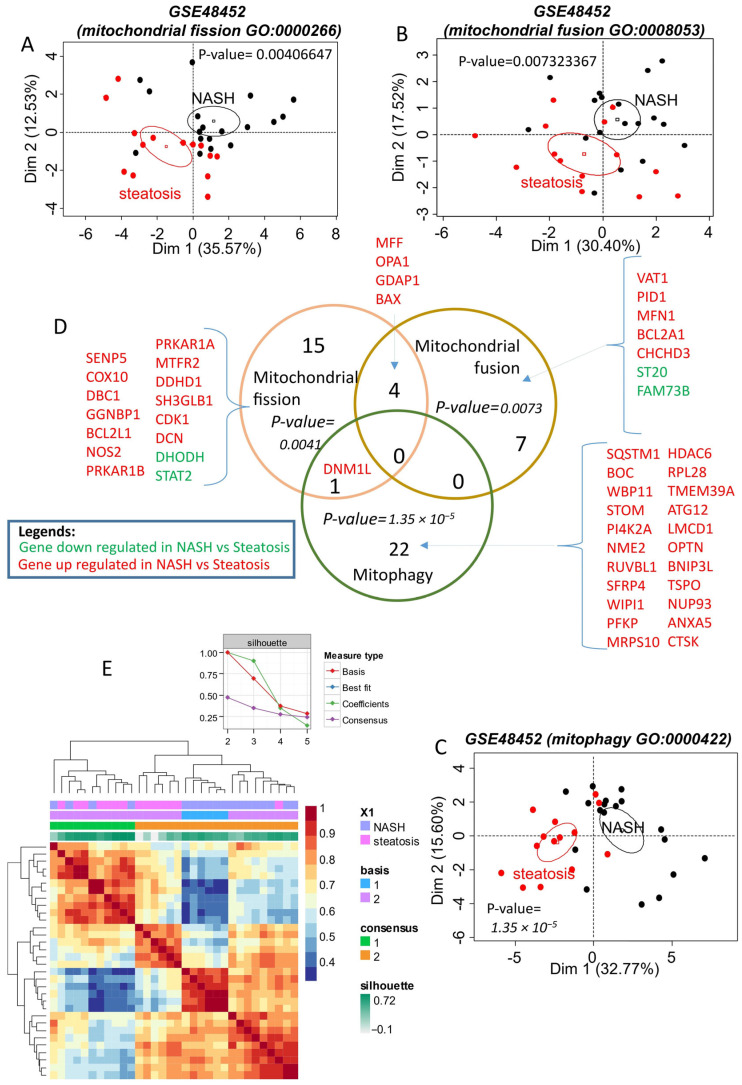
Mitochondrial turnover (fusion–fission–mitophagy) alterations in NASH (training dataset GSE48452): (**A**): unsupervised principal component analysis performed with mitochondrial fission differentially expressed genes between NASH and steatosis on training dataset GSE48452; (**B**): unsupervised principal component analysis performed with mitochondrial fusion differentially expressed genes between NASH and steatosis on training dataset GSE48452; (**C**): unsupervised principal component analysis performed with mitophagy differentially expressed genes between NASH and steatosis on training dataset GSE48452; (**D**): Venn diagram of differential expression genes. P-values for each functionality were calculated by principal component analysis; (**E**): Heatmap of the consensus matrix (100 iterations) from non-negative matrix factorization performed with the mitochondrial expression profile (fission-fusion-mitophagy) on the GSE48452 training dataset.

Analysis showed that the mitochondrial fission function, primarily represented by Gene Ontology identifier GO:0000266, was disrupted in NASH (20 genes, Supplementary Table S1). The differentially expressed fission genes significantly enabled the discrimination of NASH samples from steatosis samples by unsupervised principal component analysis (*p*-value = 0.0041, Figure 1A and Supplementary Figure S1A). Notably, the majority of fission differentially expressed genes were upregulated in NASH (up: *n* = 18; down: *n* = 2; Supplementary Figure S1B and Supplementary Table S1). Moreover, the differentially expressed genes of mitochondrial fusion in NASH versus steatosis on the training dataset GSE48452 are presented in Supplemental Figure S2.

A Venn diagram (Figure 1D) intersecting these gene lists revealed that some altered genes in NASH share functions, such as *MFF*, *OPA1*, *GDAP1*, and *BAX,* which are upregulated and associated with both fission and fusion. Similarly, *DNM1L* (encoding the Drp1 protein), which is upregulated in NASH, is involved in both fission and mitophagy. *DNM1L* is one of the genes exhibiting the greatest fold change in expression when comparing NASH and steatosis (Supplementary Table S1). The majority of mitochondrial genes altered in NASH were upregulated, with mitophagy being the most represented function (Figure 1D, Supplementary Table S1). The upregulated genes of mitophagy in NASH versus steatosis on the training dataset GSE48452 are further detailed in Supplemental Figure S3.

The association of differentially expressed genes from fission, fusion, and mitophagy functions enabled effective reclassification of the samples by unsupervised analysis. The silhouette graph of non-negative matrix factorization showed that the optimal consensus coefficient was of rank 2, corresponding to the two groups tested: NASH and steatosis. These groups were well discriminated in the consensus matrix heatmap (Figure 1E).

### 2.2. The Mitochondrial Turnover Gene Signature, Including Drp1, Is Associated with Advanced Fibrosis in NAFLD

**Figure 2:**
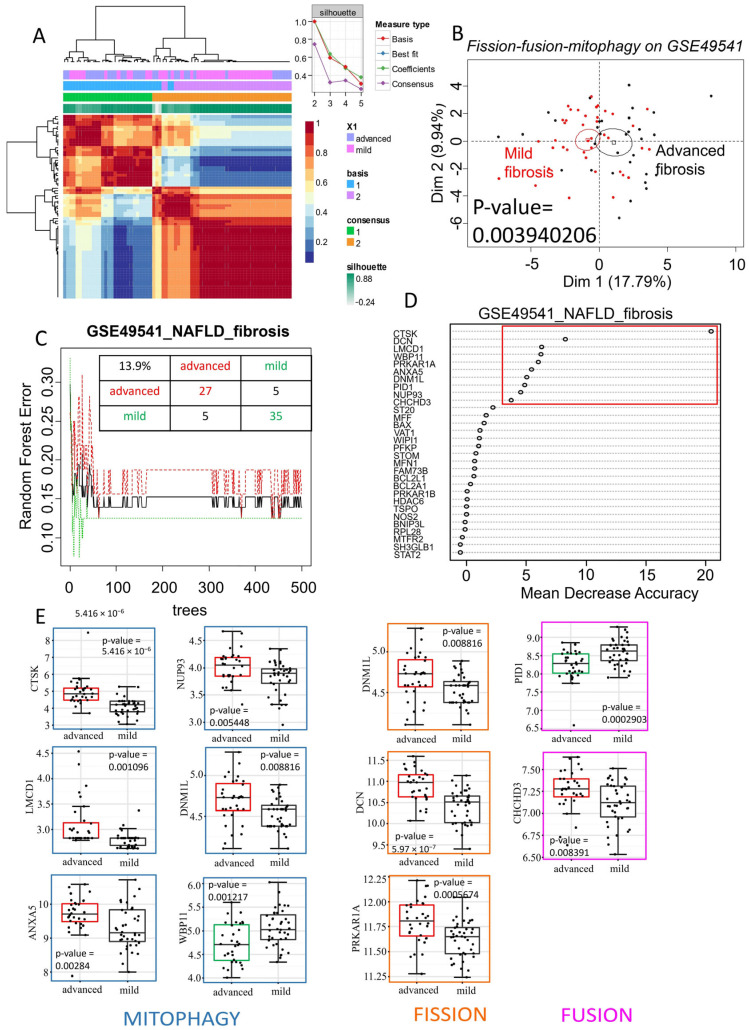
Mitochondrial turnover associated with advanced fibrosis in NAFLD: (**A**): heatmap of consensus matrix (100 iterations) from non-negative matrix factorization performed with mitochondrial expression profile (fission–fusion–mitophagy) on validation dataset GSE49541. Consensus coefficient was maximal with rank 2 of clustering matching with NAFLD with advanced fibrosis (blue) and (purple) NAFLD with mild fibrosis patient groups; unsupervised principal component analysis performed with mitochondrial expression profile between NAFLD with mild or advanced fibrosis on validation dataset GSE49541. (**B**): Representation of a principal component analysis performed with the mitochondrial expression profile (fission–fusion–mitophagy) on the validation dataset GSE49541, demonstrating a significant separation between “mild fibrosis” and “advanced fibrosis” patient samples. (**C**): Random Forest error plot created with mitochondrial expression profile on validation dataset GSE49541: a total misclassification error was evaluated at 13.9% with a learning of 500 trees. (**D**): Gene importance plot performed with Random Forest algorithm and mitochondrial gene expression profile on validation set: 10 most important discriminant genes taking account of fibrosis grades in NAFLD are highlighted in red. (**E**): Gene expression boxplots of 10 most important discriminant genes taking account of fibrosis grades in NAFLD: genes are classed by their mitochondrial function (blue: mitophagy; orange: fission; pink: fusion); green boxes represent downregulated genes in advanced fibrosis; red boxes represent upregulated genes in advanced fibrosis; *p*-values were calculated by two-sided *t*-test with Welch correction.

The microarray dataset GSE49541, which includes NAFLD patients at different stages of fibrosis, was used as an independent validation cohort to test the previously identified mitochondrial turnover gene set. The association of differentially expressed genes related to fission, fusion, and mitophagy (from the training dataset, Supplementary Table S1) also allowed for effective reclassification of the samples through unsupervised analysis in this validation cohort. The silhouette graph of non-negative matrix factorization showed that the optimal consensus coefficient was of rank 2, corresponding to the two groups tested: NAFLD with advanced fibrosis and NAFLD with mild fibrosis. These groups were well discriminated in the consensus matrix heatmap (Figure 2A). Unsupervised principal component analysis using mitochondrial turnover genes also allowed for significant discrimination between the NAFLD (advanced fibrosis) and NAFLD (mild fibrosis) groups (*p*-value = 0.0039, Figure 2B).

Random Forest analysis, performed with 500 tree iterations, estimated that mitochondrial turnover genes have an overall misclassification error rate of 13.9% between the samples in this independent validation dataset. These results suggest that mitochondrial turnover genes, previously identified as differentially expressed in NASH versus steatosis, can distinguish NAFLD samples based on their fibrosis stage. The Random Forest analysis (Figure 2C) ranked genes according to their importance for discriminating the samples. In this context, the variables are genes, and this analysis highlighted 10 key genes for distinguishing NAFLD samples based on fibrosis stage (Figure 2D). Eight of these genes were upregulated in advanced-fibrosis NAFLD, including *CTSK*, *LMCD1*, *ANXA5*, *NUP93*, *DNM1L*, *DCN*, *PRKAR1A*, and *CHCHD3*, while two genes, *WBP11* and *PID1*, were downregulated (Figure 2E). Most of these genes were associated with mitophagy, followed by fission, and then fusion. Notably, *DNM1L*, which is involved in both mitophagy and fission functions, was found to be upregulated in advanced-fibrosis NAFLD compared to mild-fibrosis NAFLD (*p*-value = 0.0088, Figure 2E). Therefore, the overexpression of Drp1, encoded by *DNML1*, appears to be a marker not only distinguishing NASH from steatosis but also indicating the progression of fibrosis.

### 2.3. Clinical Validation of Drp1 Expressions

**Figure 3:**
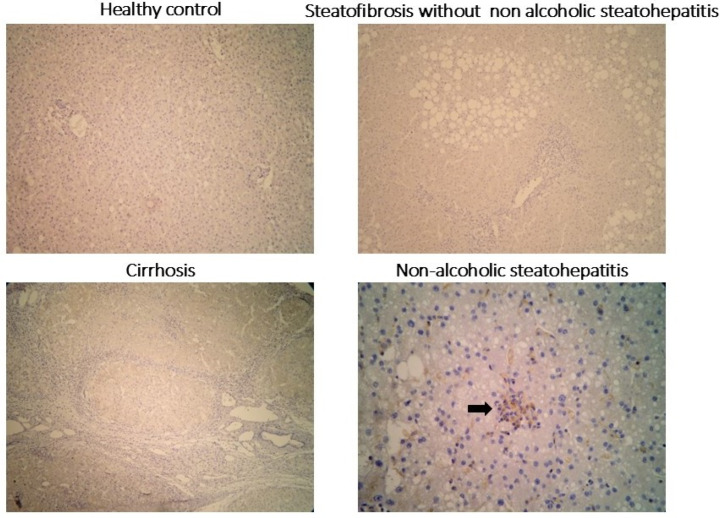
Immunohistochemical staining for Drp1 in liver biopsies diagnosed as normal, steatotic, cirrhotic, and non-alcoholic steatohepatitis (NASH): Kupffer cells within lobular inflammatory infiltrates (arrow) exhibit positive Drp1 expression, whereas the liver parenchyma expression remains negative in all conditions.

Since Drp1 was identified as a key upregulated gene in the in silico analysis, particularly in advanced-fibrosis NAFLD, its expression was further investigated in an independent cohort of human liver tissue samples in our institution. This cohort included five normal livers, five livers with steatosis, five livers with NASH, and five livers with NASH-related cirrhosis. Drp1 expression was evaluated using immunohistochemistry (Figure 3). No Drp1 expression was observed in steatotic tissue, while its expression progressively increased with disease severity, peaking in cirrhosis. Notably, Drp1 was specifically localized to Kupffer cells, and its abundance directly correlated with the degree of fibrosis.

### 2.4. Discussion

NAFLD has emerged as the most common chronic liver disease, with alarming projections indicating a 55% worldwide prevalence by 2040 [13]. Up to one-third of NAFLD patients will develop NASH, and around 40% of NASH patients will experience fibrosis progression [14]. However, there are currently few reliable tools to predict which patients will progress in the disease, and no pharmacological agents are available to treat NASH, highlighting the need for further elucidation of the underlying mechanisms. Disruption in the balance between mitochondrial fusion and fission is thought to play a role in various pathological conditions, including neurodegenerative, neoplastic, endocrine, and cardiovascular diseases [15,16,17]. This study investigated genes involved in mitochondrial dynamics during NAFLD progression, uncovering differential expressions in 44 mitochondrial genes associated with fission, fusion, and mitophagy, particularly linked to the shift from steatosis to NASH. Additionally, 10 genes were identified with differential expression during the progression of hepatic fibrosis in NASH. The study particularly emphasized the role of Drp1 (*DMNL1*) in NAFLD progression, with elevated expression of Drp1 observed in human liver tissues affected by inflammation and fibrosis, particularly in Kupffer cells.

Mitochondrial dynamics serve to both satisfy the high energy requirements of hepatocytes and ensure the elimination of damaged mitochondria during metabolic stress, such as that observed in NAFLD [18]. It appears that, when a cell is in a delicate balance between survival and death, the mitochondrion acts as a decisive factor in determining the outcome. While pro-survival signals may preserve the mitochondrial membrane and allow the organelle to continue producing energy, this carries the risk of damaged mitochondria producing ROS, which in turn can drive inflammation, repair, or cell death pathways [19]. Increasing evidence suggests that excessive mitochondrial fission is linked to oxidative stress and impaired mitochondrial function [20,21,22]. Exposure to palmitate, a long-chain saturated fatty acid known for its lipotoxic properties, has been reported to induce mitochondrial fragmentation in HepG2 cells, leading to elevated superoxide levels and oxidative stress [20]. Moreover, Galloway et al. reported that mice fed a high-fat diet exhibited fragmented mitochondria and metabolic dysfunction, consistent with NAFLD [21]. Remarkably, transgenic inhibition of mitochondrial fission in these mice suppressed both hepatic oxidative stress and steatosis [22]. On the other hand, defective mitochondrial fusion regulation may also play a role in the onset of steatohepatitis. In NAFLD mouse models, a reduction in Mitofusin 1 (MFN1), a key protein mediating mitochondrial fusion, is consistently observed [23,24]. Similarly, exposure of hepatocytes to palmitate results in decreased transcript and protein levels of Mitofusin 2 (MFN2), another vital regulator of mitochondrial dynamics and endoplasmic reticulum–mitochondria interactions [20]. This decline in MFN2 levels is also observed in liver samples from both NASH patients and murine NAFLD models [25,26]. Notably, hepatic deletion of *MFN2* in mice drastically worsens inflammation, triglyceride accumulation, fibrosis, and hepatocellular carcinoma (HCC) in NASH models [27]. Conversely, restoring MFN2 expression through adenoviral delivery in liver-specific MFN2-deficient mice effectively mitigates NASH-related symptoms [28].

Disruptions in mitochondrial homeostasis can negatively impact hepatocyte survival and advance NASH progression; however, identifying reliable biomarkers to accurately reflect these defects remains a challenge. Drp1 is a cytosolic protein central to the regulation of both mitochondrial fission and the process of mitophagy [29]. This study highlights that *DNM1L*, encoding for Drp1, is one of the genes showing the greatest fold change in expression when comparing NASH and steatosis, and it was upregulated in advanced-fibrosis NAFLD compared to mild-fibrosis NAFLD. The study also observed that Drp1 protein overexpression in hepatic tissue correlates with NAFLD progression. Prior research in cell cultures and mouse models suggests that disruptions in Drp1-mediated mitochondrial fission are linked to mitochondrial dysfunction and liver cell damage [21,22]. Using high-fat-diet-induced NAFLD as a model, increased protein levels of Drp1, mitochondrial fragmentation, and heightened hepatocyte lipolysis are observed in liver [21]. Moreover, inhibition of mitochondrial division through expressing the dominant-negative fission mutant (Drp1-K38A) alleviates the oxidative stress and impairment of liver function resulting from excess intake of fat, exerting protective effects against liver steatosis [22].

This study reveals that Drp1 is predominantly expressed in Kupffer cells within lobular inflammatory infiltrates, indicating a correlation between hepatic Drp1 expression and the amount of Kupffer cells in the tissue. In Kupffer cells, Drp1 is essential in mitochondria to promote the continued clearance of apoptotic cells, a process known as efferocytosis [30,31]. This process prevents post-apoptotic necrosis, reduces inflammation, and promotes pro-resolving responses in phagocytes [32,33]. Wang et al. demonstrated that the uptake of multiple apoptotic cells by macrophages requires Drp1-mediated mitochondrial fission, which is triggered by apoptotic cell uptake [32]. Most importantly, when mitochondrial fission was prevented in macrophages exposed to a first round of apoptotic cells, subsequent apoptotic cell uptake was compromised. Additionally, in Kupffer cells, Drp1 plays a critical role in LPS-induced liver injury by mediating mitochondrial fission and subsequent release of mitochondrial DNA (mtDNA) into the cytosol [34]. Drp1-dependent mtDNA release activates the STING signaling pathway, thereby promoting ROS production and hepatocyte death during sepsis. These results highlight Drp1 as a key therapeutic target for preventing liver damage. Promisingly, inhibiting Drp1 with Mdivi-1 modulates ROS and may protect liver function [35,36]. Moreover, modulation of impaired efferocytosis driven by Drp1 in advanced lesions has emerged as a promising strategy. This can be achieved by targeting inflammation-induced cleavage of the macrophage efferocytosis receptor MerTK with tyrosine kinase inhibitors [37], or by using astaxanthin, a non-provitamin A carotenoid, through modulation of ERK2, COX-2, and NF-κB [38].

## 3. Material and Methods

### 3.1. Microarray Datasets

Two datasets available online at the NCBI-GEO (http://www.ncbi.nlm.nih.gov/geo/, accessed on 27 July 2025) were downloaded. The GSE48452 dataset [39] was chosen as the training set, comprising array-based expression profiles of 33,297 genes across 73 samples which were grouped into C (control = 14), H (healthy obese = 27), S (steatosis = 14), and N (NASH = 18). The GSE49541 dataset [40] was selected as the validation set, containing a total of 72 RNA profiles from liver samples, including 40 belonging to mild NAFLD (fibrosis stage 0–1) and 32 belonging to advanced NAFLD (fibrosis stage 3–4).

### 3.2. Bioinformatics

Bioinformatics and biostatistics analyses were performed using the R software environment, version 3.2.3. Student’s *t*-tests were conducted with two-sided hypotheses and a 5% error threshold, applying Welch’s correction to account for unequal variances. Heatmap visualizations were generated using the heatplot function from the R package *made4* [41].

### 3.3. Genesets Associated with Mitochondrial Functionalities

Determination of genes associated with mitochondrial functions was realized by Pubmed curation and querying the Gene Ontology database, implemented on website AmiGO2 (http://amigo.geneontology.org/amigo/landing, accessed on 27 July 2025), by employing the keywords mitochondrial fission, mitochondrial fusion and mytophagy, associated with the following Gene Ontology identifiers: GO:0000266, GO:0008053 and GO:0000422, respectively [42].

### 3.4. Differentially Expressed Genes

Differential expression gene analysis for NASH compared to steatosis in the training dataset was performed using the Significance Analysis of Microarrays (SAM) algorithm with a false discovery rate (FDR) of less than 5% [43]. Mitochondrial differentially expressed genes identified as being associated with three mitochondrial functions (mitochondrial fission, mitochondrial fusion, and mitophagy) were collected to create a mitochondrial turnover expression profile, which was then tested on independent validation sets.

### 3.5. Non-Negative Matrix Factorization

To perform gene set validation by unsupervised classification on the independent cohort, the non-negative matrix factorization (NMF) algorithm was employed in R software, implemented in the R package NMF [44]. Using mitochondrial differentially expressed genes, 100 iterations were performed with classification ranks ranging from 2 to 5. The choice of rank classification was based on determining the maximal consensus coefficient, after which the rank started to decrease. Finally, NMF was performed again with 100 iterations on Euclidean distances and the previously determined optimal rank. Classification results were graphically represented by a heatmap of the consensus matrix, evaluated over the 100 iterations.

### 3.6. Random Forest

Misclassification error on validation datasets (independent cohort) was assessed using the Random Forest algorithm, implemented in the RandomForest R package. First, the minimum error of the bagging procedure was estimated using the RFtune function to determine the optimal “mtry” parameter. Second, the Random Forest algorithm was applied with the optimal “mtry” parameter on a forest of 500 trees [45].

### 3.7. Principal Component Analysis

Unsupervised principal component analysis was performed using the FactoMineR R package. *p*-values for group discrimination were calculated with the dimdesc function on the categorical group variable, which was fitted to the principal components [46].

### 3.8. Human Tissue

Human liver samples analyzed for Drp1 expression by immunohistochemistry were biopsies obtained during graft harvesting from donors or liver surgery, and histological analysis was performed by an experienced pathologist (MS). Our institutional review board (Paul Brousse hospital-Centre des Ressources Biologiques Paris-SUD, CRB Paris Sud, Bio Banking Number: 0033-00089) approved the study conduct. Patient non-opposition was obtained according to French ethical laws regarding non-interventional research. The study was conducted in accordance with the relevant “Declaration of Helsinki” and “International Conference on Harmonization Good Clinical Practice” guidelines.

### 3.9. Immunohistochemistry

Tissues were cut into slices with a 7 μm thickness, and cryosections were fixed in acetone or 4% paraformaldehyde. Following blocking in 4% serum, sections were incubated in primary antibodies (Drp1 for human IHC (1:50, Abcam #ab56788, Cambridge, MA, USA)) and then incubated with a biotin-labeled secondary antibody (Vector Laboratories, Burlingame, CA, USA), followed by incubation with streptavidin-peroxidase (KPL, Gaithersburg, MD, USA) and then incubation in AEC solution (Dako, Glostrup, Denmark).

## 4. Conclusions

Overall, impaired expression of genes related to mitochondrial dynamics appears to be associated with the transition from steatosis to NASH and fibrosis. Specifically, the increased expression of Drp1 during NAFLD may signal the onset of inflammation and fibrosis in NASH. This increase could enhance the role of Drp1 in maintaining mitochondrial dynamics by modulating mitochondrial morphology and facilitating the clearance of apoptotic cells. When macrophages become impaired in their ability to engulf multiple dead cells, elevated Drp1 levels may directly contribute to inflammation and fibrosis. The study points out that Drp1 could serve as both a marker for the progression from steatosis to NASH and for the increasing stages of fibrosis, as well as a potent indicator of Kupffer cell infiltration in the liver, indicating that Drp1 could be a valuable therapeutic target for the treatment of NASH and liver fibrosis.

## Data Availability

The data presented in this study are available upon request from the corresponding author.

## Supplementary Materials

The supporting information can be downloaded at https://www.mdpi.com/article/10.3390/ijms26157373/s1.

## Abbreviations

Drp1: dynamin-related protein 1; FDR: false discovery rate; GC-RMA: GeneChip Robust Multi-array Average; MFN1: mitofusin 1; MFN2: mitofusin 2; mtDNA: mitochondrial DNA; NAFLD: non-alcoholic fatty liver disease; NASH: non-alcoholic steatohepatitis; NMF: non-negative matrix factorization; ROS: reactive oxygen species; SAM: Significance Analysis of Microarrays.
