# Supplementary material for: Analysis of the Mitochondrial Dynamics in NAFLD: Drp1 as a Marker of Inflammation and Fibrosis
Source: Int J Mol Sci. 2025 Jul 30;26(15):7373. doi: 10.3390/ijms26157373 (PMC12347318; doi:10.3390/ijms26157373)
Supplement: Supplementary file 1 [file ijms-26-07373-s001.zip › Supplemental figures.pdf]

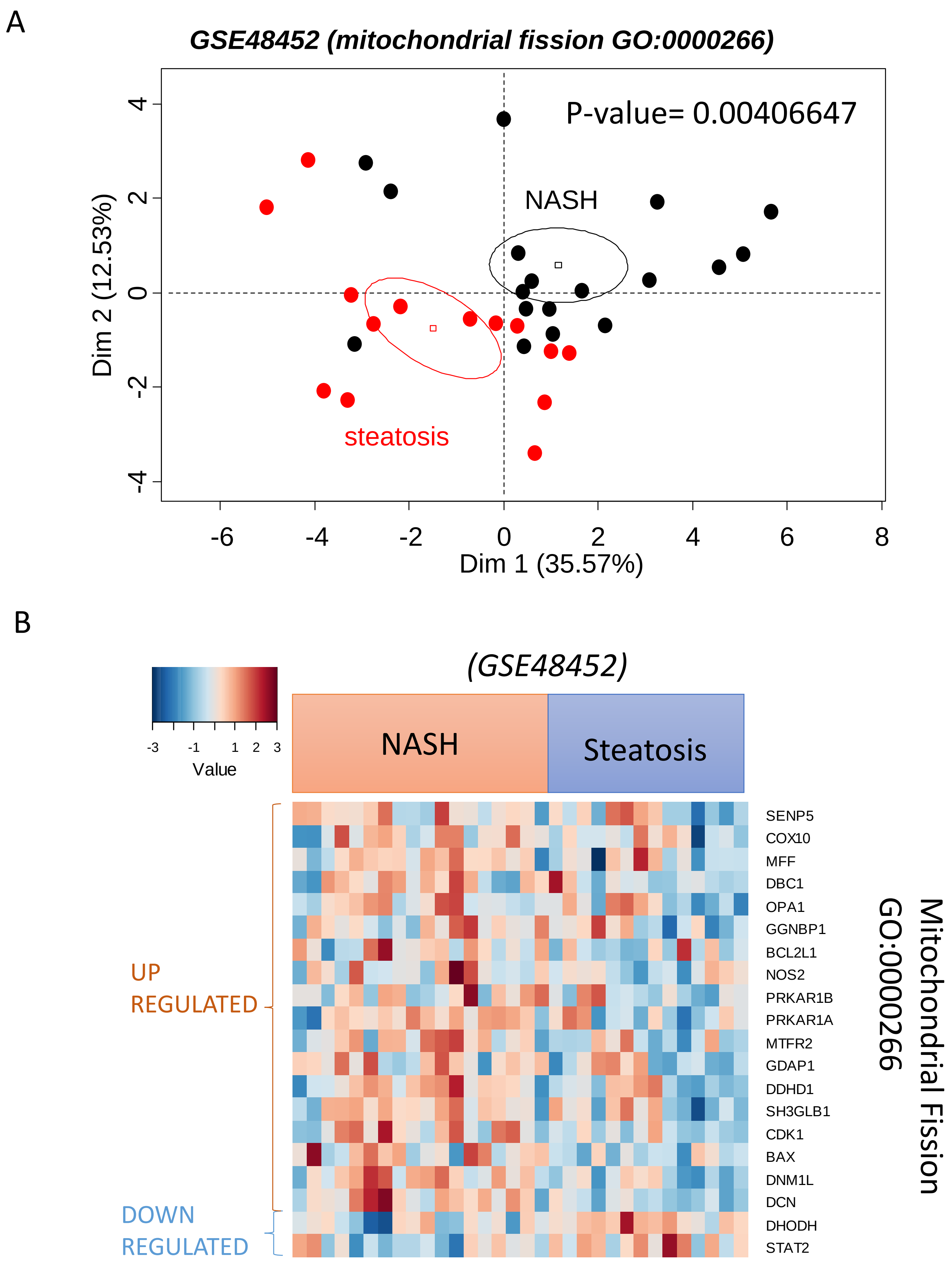

Supplemental figure S1: differentially expressed genes of mitochondrial fission in NASH versus Steatosis on training dataset GSE48452

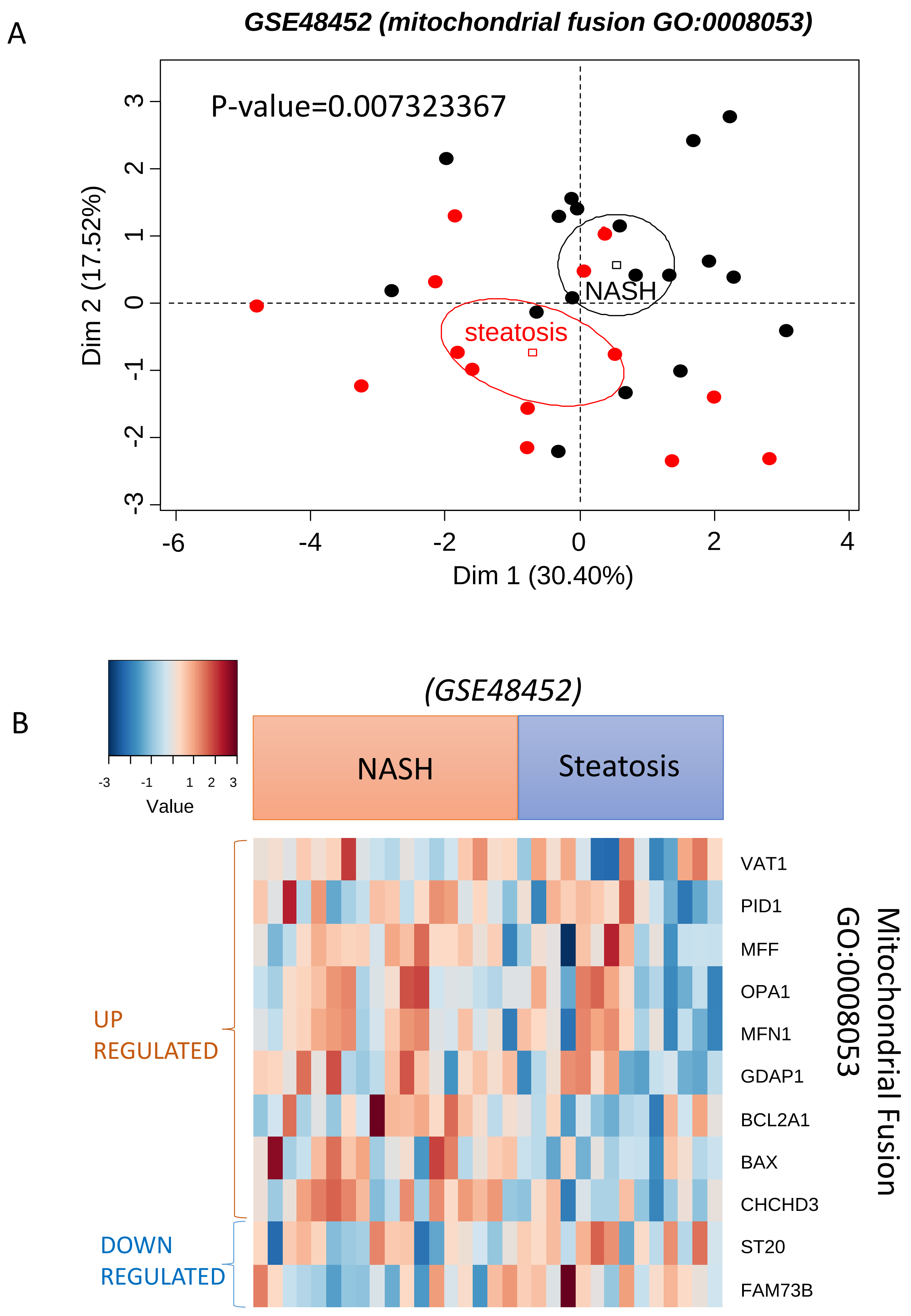

Supplemental figure S2: differentially expressed genes of mitochondrial fusion in NASH versus Steatosis on training dataset GSE48452

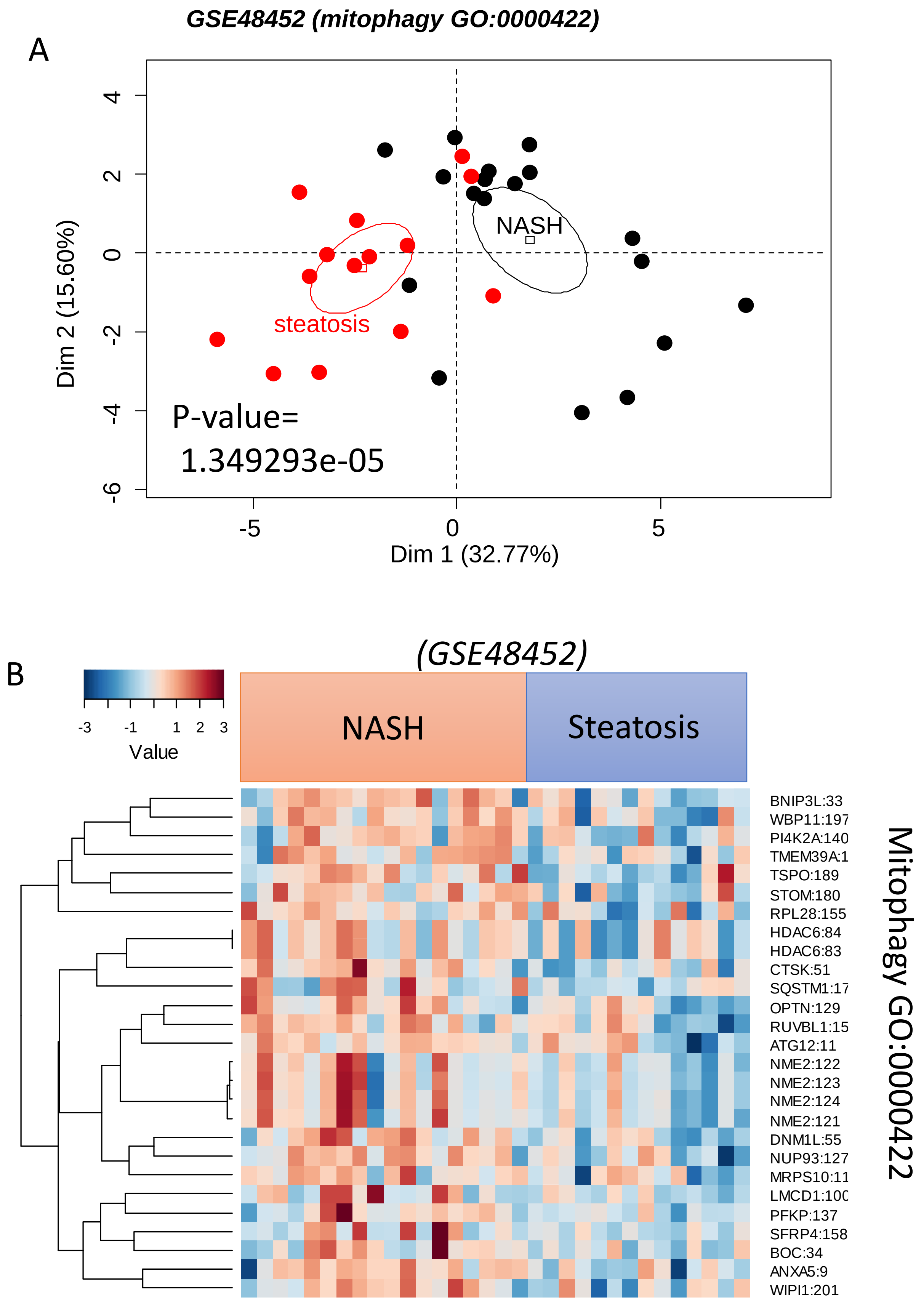

Supplemental figure S3: up regulated genes of mitophagy in NASH versus Steatosis on training dataset GSE48452
